# Yeast Elongator protein Elp1p does not undergo proteolytic processing in exponentially growing cells

**DOI:** 10.1002/mbo3.285

**Published:** 2015-09-25

**Authors:** Hao Xu, Joakim Bygdell, Gunnar Wingsle, Anders S. Byström

**Affiliations:** ^1^ Department of Molecular BiologyUmeå University901 87UmeåSweden; ^2^ Department of Forest Genetics and Plant PhysiologyUmeå Plant Science CentreSwedish University of Agricultural Sciences901 87UmeåSweden; ^3^ Computational Life Science Cluster (CLiC)Department of ChemistryUmeUmeå University901 87UmeåSweden

**Keywords:** Elongator complex, Elp1p, Prb1p, proteolysis, *Saccharomyces cerevisiae*, tRNA modification

## Abstract

In eukaryotic organisms, Elongator is a six‐subunit protein complex required for the formation of 5‐carbamoylmethyl (ncm^5^) and 5‐methylcarboxymethyl (mcm^5^) side chains on uridines present at the wobble position (U_34_) of tRNA. The open reading frame encoding the largest Elongator subunit Elp1p has two in‐frame 5′ AUG methionine codons separated by 48 nucleotides. Here, we show that the second AUG acts as the start codon of translation. Furthermore, Elp1p was previously shown to exist in two major forms of which one was generated by proteolysis of full‐length Elp1p and this proteolytic cleavage was suggested to regulate Elongator complex activity. In this study, we found that the vacuolar protease Prb1p was responsible for the cleavage of Elp1p. The cleavage occurs between residues 203 (Lys) and 204 (Ala) as shown by amine reactive Tandem Mass Tag followed by LC‐MS/MS (liquid chromatography mass spectrometry) analysis. However, using a modified protein extraction procedure, including trichloroacetic acid, only full‐length Elp1p was observed, showing that truncation of Elp1p is an artifact occurring during protein extraction. Consequently, our results indicate that N‐terminal truncation of Elp1p is not likely to regulate Elongator complex activity.

## Introduction

The Elongator complex of *Saccharomyces cerevisiae* was first reported to be associated with the hyper phosphorylated elongating form of RNA polymerase II (Pol II) and three proteins (Elp1p, Elp2p, and Elp3p) constituted the identified complex (Otero et al. 1999). Subsequently, Elp4, Elp5, and Elp6 were identified to be a subcomplex of Elongator complex (Krogan and Greenblatt 2001; Li et al. 2001; Winkler et al. 2001). Initially, the complex was suggested to be involved in elongation of Pol II transcription through histone H3 and H4 acetylation (Wittschieben et al. 1999). Additional studies reported a role of Elongator in other cellular processes, that is, polarized exocytosis (Rahl et al. 2005), DNA repair (Li et al. 2009), and formation of 5‐methoxycarbonylmethyl (mcm^5^) or 5‐carbamoylmethyl (ncm^5^) side chains at the wobble position (U_34_) (Huang et al. 2005).

In yeast, 11 tRNA species have a mcm^5^ or ncm^5^ side chains at the wobble position and three of these species, ${\text{tRNA}}_{{\mathrm{mcm}}^{5}s^{2}\mathrm{UUU}}^{\mathrm{Lys}}$, ${\text{tRNA}}_{{\mathrm{mcm}}^{5}s^{2}\mathrm{UUG}}^{\mathrm{Gln}}$, and ${\text{tRNA}}_{{\mathrm{mcm}}^{5}s^{2}\mathrm{UUC}}^{\mathrm{Glu}}$ are also modified with a 2‐thio group, generating the modified nucleoside 5‐methoxycarbonylmethyl‐2‐thiouridine (mcm^5^s^2^U) (Smith et al. 1973; Kobayashi et al. 1974; Kuntzel et al. 1975; Yamamoto et al. 1985; Keith et al. 1990; Glasser et al. 1992; Huang et al. 2005; Lu et al. 2005; Johansson et al. 2008). Overexpression of various combinations of hypomodified ${\text{tRNA}}_{s^{2}\mathrm{UUU}}^{\mathrm{Lys}}$, ${\text{tRNA}}_{s^{2}\mathrm{UUG}}^{\mathrm{Gln}}$, and ${\text{tRNA}}_{s^{2}\mathrm{UUC}}^{\mathrm{Glu}}$ suppresses the Elongator‐dependent phenotypes in Pol II transcription, exocytosis, and DNA repair, but not the tRNA modification defect (Esberg et al. 2006; Chen et al. 2011). Thus, the physiological relevant function of Elongator complex in yeast is in formation of mcm^5^ and ncm^5^ side chains at U_34_ of tRNA (Esberg et al. 2006; Chen et al. 2011). This hypothesis was recently supported by the observation that *Min*Elp3, the homolog of yeast Elp3p in the archaea *Methanocaldococcus infernus*, produced cm^5^U in the presence of SAM (*S*‐adenosyl methionine) and acetyl‐CoA (Selvadurai et al. 2014). As presence of wobble uridine modifications are important for efficient translation in yeast, the pleiotropic phenotypes of mutants deficient in wobble uridine modifications seem to be caused by a defect in translation (Esberg et al. 2006; Björk et al. 2007; Dewez et al. 2008; Johansson et al. 2008; Nakai et al. 2008; Schlieker et al. 2008; Leidel et al. 2009; Chen et al. 2011; Bauer and Hermand 2012; Bauer et al. 2012; Rezgui et al. 2013; Zinshteyn and Gilbert 2013).

Elp1p, the largest subunit of the Elongator complex, is a phosphoprotein and its dephosphorylation was dependent on the phosphatase Sit4p and its associated partners – Sap185p and Sap190p (Jablonowski et al. 2004). In a *sit4* null mutant, Elp1p is hyperphosphorylated, whereas in the casein kinase *hrr25* null mutant, Elp1p was hypophosphorylated (Mehlgarten et al. 2009). The proportion of hyper‐ and hypophosphorylated Elp1p is balanced in wild‐type cells and any changes that perturb this equilibrium was suggested to result in inactivation of the Elongator (Mehlgarten et al. 2009). Therefore, Sit4p and Hrr25p seem to regulate the phosphorylation status of Elp1p and play antagonistic roles in the function of the Elongator complex (Mehlgarten et al. 2009). In a recent study, nine in vivo phosphorylation sites within Elp1p were identified and Hrr25p directly phosphorylates two of them (Ser‐1198 and Ser‐1202) (Abdel‐Fattah et al. 2015). These authors concluded that Elp1p phosphorylation plays a positive role in tRNA modification.

Aside from phosphorylation, Elp1p also undergoes proteolysis. Affinity purification of Elongator from a strain having a carboxy terminal tandem affinity purification (TAP) tag or western blot analysis of strains having an Elp1p tagged with human influenza hemagglutinin (HA), revealed two major and a minor form of Elp1p (Krogan and Greenblatt 2001; Fichtner et al. 2003). LC‐MS (liquid chromatography mass spectrometry) analysis showed that the shortest form had an N‐terminal truncation, resulting in a removal of about 200 amino acids (Fichtner et al. 2003). In mutants lacking Urm1p or Kti11p, the level of the N‐terminal‐truncated Elp1p increased (Fichtner et al. 2003). The *URM1* and *KTI11* gene products were linked to Elongator as strains with these genes mutated as well as Elongator mutants are resistant to zymocin, a *Kluyveromyces lactis* toxin (Frohloff et al. 2001; Fichtner and Schaffrath 2002; Huang et al. 2008). Now it is known that the *γ*‐toxin, a subunit of zymocin, is an endonuclease that targets ${\text{tRNA}}_{{\mathrm{mcm}}^{5}s^{2}\mathrm{UUC}}^{\mathrm{Glu}}$, ${\text{tRNA}}_{{\mathrm{mcm}}^{5}s^{2}\mathrm{UUG}}^{\mathrm{Glu}}$, and ${\text{tRNA}}_{{\mathrm{mcm}}^{5}s^{2}\mathrm{UUU}}^{\mathrm{Lys}}$ (Lu et al. 2005). At the wobble position, these tRNAs have the modified nucleoside mcm^5^s^2^U_34_ and the endonuclease cleaves these tRNAs between U_34_ and U_35_ provided that the wobble nucleoside is fully modified (Lu et al. 2005). The Kti11p is required for formation of the mcm^5^ and Urm1p for the s^2^ group of the mcm^5^s^2^U_34_ nucleoside (Huang et al. 2008). Loss of Urm1p or Kti11p increases the amount of truncated Elp1p, abolishes formation of mcm^5^ or s^2^ group of mcm^5^s^2^U_34_, and therefore makes cells resistant to *γ*‐toxin, suggesting that both Kti11p and Urm1p influenced Elp1p proteolysis and are required for proper Elongator function/regulation (Fichtner et al. 2003). However, it was not established how the truncated form of Elp1p was generated, neither was the exact truncation site determined.

In this study, we identified the vacuolar protease Prb1p to be required for cleavage of Elp1p between its 203rd (Lys) and 204th (Ala) residues. Expression of N‐terminal truncated Elp1p did not complement the wobble uridine tRNA modification defect of strain with an *elp1Δ* null allele. We found that appearance of N‐terminal truncated Elp1p is a preparation artifact which can be circumvented using an alternative protein extraction method.

## Experimental Procedures

### Strains, medium, and genetic procedure

Yeast transformation, media, and genetic procedures have been described elsewhere (Burke et al. 2000). Strains used in the peptidase/protease screen were from the yeast knock out MATa collection (Open Biosystems, Inc., Lafayette, Indiana, USA, Cat. YSC1053) (Table S1). The *ELP1‐TAP‐HIS3* strain (YSC1177‐YLR384C) was purchased from the Open Biosystems TAP‐tagged open reading frame (ORF) collection. In the YSC1177‐YLR384C strain, the level of mcm^5^s^2^U in total tRNA is 96.8% of the wild‐type strain, showing that Elp1‐Tap construct is functional (data not shown). Strain YSC1177‐YLR384C was mated with BY4742, the diploid was sporulated and tetrad dissection generated the MAT*α* strain derivative *ELP1‐TAP‐HIS3*, UMY3692. Strain UMY3692 was mated with the *prb1::kanMX* null allele‐containing strain YSC1053‐YEL060C (Open Biosystems, Inc.) to generate strain UMY3864 in which the *ELP1‐TAP‐ HIS3* and *prb1::kanMX* alleles are combined. The *prb1::kanMX* strain, YSC1053‐YEL060C, was mated with strain BY4742 to generate the *MATα* strain derivative *prb1::kanMX*, UMY3863. Diploid *elp1Δ/elp1Δ* (YSC1056‐YLR384C, Yeast Homozygous Diploid Collection) was sporulated and tetrad dissected to obtain the S288C *elp1::KanMX* null mutant, UMY3906. The *ELP1‐GFP‐HIS3MX6* strain 95700‐YLR384C (Invitrogen, Carlsbad, California, USA) was crossed with UMY3863 to combine the *prb1::kanMX*and *ELP1‐GFP‐HIS3MX6* alleles generating strain UMY3930. In strain 95700‐YLR384C, the level of mcm^5^s^2^U in total tRNA is 60.5% of the wild‐type strain (data not shown). Strains used in this study are listed in Table 1.

**Table 1:** Yeast strains used in this study

Strains	Genotype	Source
BY4741	*MATa his3Δ1 leu2Δ0 ura3Δ0 met15Δ0*	Brachmann et al. (1998)
BY4742	*MATα his3Δ1 leu2Δ0 ura3Δ0 lys2Δ0*	Brachmann et al. (1998)
YSC1177‐YLR384C	*MATa his3Δ1 leu2Δ0 ura3Δ0 met15Δ0 ELP1::TAP‐HIS3MX6*	Open Biosystems, Inc.
UMY3692	*MATα his3Δ1 leu2Δ0 ura3Δ0 lys2Δ0 ELP1::TAP‐HIS3MX6*	This study
YSC1053‐YEL060C	*MATa his3Δ1 leu2Δ0 ura3Δ0 met15Δ0 prb1::kanMX4*	Open Biosystems, Inc.
UMY3863	*MATα his3Δ1 leu2Δ0 ura3Δ0 met15Δ0 prb1::kanMX4*	This study
UMY3864	*MATa his3Δ1 leu2Δ0 ura3Δ0 met15Δ0 prb1::kanMX ELP1::TAP‐HIS3*	This study
YSC1056‐YLR384C	*elp1Δ/elp1Δ his3Δ1/his3Δ1 leu2Δ0/ leu2Δ0 ura3Δ0/ura3Δ0 met15Δ0/MET15 lys2Δ0/LYS2*	Open Biosystems, Inc.
UMY3906	*MATa his3∆1 leu2∆0 ura3∆0 elp1::kanMX4*	This study
95700‐YLR384C	*MATa his3Δ1 leu2Δ0 ura3Δ0 met15Δ0 ELP1::GFP‐HIS3MX6*	Invitrogen
UMY3930	*MATa his3Δ1 leu2Δ0 ura3Δ0 met15Δ0 ELP1::GFP‐HIS3MX6 prb1::kanMX4*	This study
UMY3551	*MATa his3Δ1 leu2Δ0 lys2Δ0 ura3Δ0 urm1::kanMX6*	This study
UMY3771	*MATa his3Δ1 leu2Δ0 lys2Δ0 ura3Δ0 kti11::kanMX6*	This study

### Plasmid construction, PCR mutagenesis, and overlapping PCR

Plasmid pBY1767, a pRS315 derivative with a functional *ELP1* gene cloned as a *Sal*I and *Sac*I fragment, was digested with restriction endonucleases *Sal*I and *Bam*HI. The *Bam*HI is unique in the *ELP1* gene and the *Sal*I/ *Bam*HI fragment containing the N‐terminus of the *ELP1* gene was cloned into the corresponding restriction sites of pRS315 generating pBY2015 (pRS315‐*ELP1*_N‐terminal_). By PCR (polymerase chain reaction)‐based mutagenesis (QuikChange^®^ Agilent Technologies, Santa Clara, California, USA, XL Site‐Directed Mutagenesis Kit, Catalog #200516), using oligonucleotides 5′‐CAGTACAAATGCCTAATGGCTTTTGGTTGAACATGACAAGA‐3′ or 5′‐GGTCAAAGAGGCAGGAGCTAAGATCAAATTTGCGTAATCTTATTA‐3′, the first (ATG_1_) or the second (ATG_2_) methionine codons of the *ELP1* ORF in plasmid pBY2015 were changed to (TTG) leucine codons, generating plasmids pRS315‐*elp1*_N‐terminal (ATG1‐TTG)_ and pRS315‐*elp1*_N‐terminal (ATG2‐TTG)_, respectively. Sequencing confirmed that no additional mutations than the ATG to TTG mutations were obtained during PCR mutagenesis. Correct plasmids were digested with restriction endonucleases *Sal*I and *Bam*HI and the fragments were cloned into plasmid pBY1767, exchanging the N‐terminal region of the *ELP1* gene, generating plasmids pBY2016 (pRS315‐*elp1*_(ATG1‐TTG)_) and pBY2017 (pRS315‐*elp1*_(ATG2‐TTG)_), respectively. To generate a plasmid expressing a truncated Elp1p starting at Ala204, overlapping PCR was applied. Plasmid pBY2015 (pRS315‐*ELP1*_N‐terminal_) was used as template and oligonucleotides 5′‐CGAGGTCGACGCTCTCCCTT‐3′ and 5′‐TTACCTACCAAACCTGATGCCATATTTGATCTTAGCTCCT‐3′ were used to amplify the promotor region and the N‐terminal part of *ELP1* including ATG_2_. Oligonucleotides 5′‐TAGTGGATCCATTTGTGATT‐3′ and 5′‐AGGAGCTAAGATCAAATATCGCATCAGGTTTGGTAGGTAA‐3′ were used to amplify DNA between the region of the *ELP1* gene encoding amino acid 204 and the unique *Bam*HI site. The PCR products were mixed and used as template for a second PCR to generate a DNA fragment where the translational start (ATG_2_) is linked to the GCA‐Ala codon corresponding to amino acid 204 in Elp1p. The PCR product was digested with restriction enzymes *Sal*I/ *Bam*HI and the fragment was used to replace the corresponding fragment of plasmid pBY1767, generating plasmid pBY2025 (pRS315‐*elp1*_ATG2‐GCA(Ala204)_). The plasmid was digested with restriction enzymes *Sac*I and *Sal*I and the *elp1*_ATG2‐GCA(Ala204)_ containing fragment was cloned into the corresponding sites of pRS425, generating pBY2050 (pRS425‐*elp1*_ATG2‐GCA(Ala204)_). Plasmids used in this study are listed in Table 2.

**Table 2:** Plasmids used in this study

Plasmids	Genotype	Source
pBY1767	pRS315‐*ELP1*	This study
pBY2015	pRS315‐*elp1*_N‐terminal_	This study
pBY2016	pRS315‐*elp1*_(ATG1‐TTG)_	This study
pBY2017	pRS315‐*elp1*_(ATG2‐TTG)_	This study
pBY2025	pRS315‐*elp1*_ATG2::GCA(Ala204)_	This study
pBY2050	pRS425‐*elp1*_ATG2::GCA(Ala204)_	This study

### tRNA isolation and high‐pressure liquid chromatography analysis

Approximately 2 g of cells was collected from yeast cultures grown to mid‐log phase and cells were resuspended in 3 mL of 0.9% NaCl. The cell suspension was vortexed with 8 mL water‐saturated phenol at room temperature (RT) for 30 min and vortexed for another 15 min with 400 μL chloroform. The water phase was isolated after centrifugation at 12,000*g* for 20 min and mixed with 4 mL phenol and vortexed for another 15 min. The water phase was collected after centrifugation at 12,000*g* for 20 min, mixed with 2.5 volumes 99.5% EtOH, and kept at −20°C for at least 3 h. Total tRNA was precipitated by centrifugation at 12,000*g* for 20 min. The pellet was dissolved in 5 mL DE52‐binding buffer (0.1 mol/L Tris‐HCl, pH 7.4, and 0.1 mol/L NaCl) and loaded onto a diethylaminoethyl DE52 cellulose column. The column was washed twice with 7 mL DE52‐binding buffer. Total tRNA was eluted with 7 mL tRNA elution buffer (0.1 mol/L Tris‐HCl, pH 7.4, and 1 mol/L NaCl) and precipitated with 5 mL isopropanol at −20°C for at least 3 h. Total tRNA was collected by a 12,000*g* centrifugation for 20 min and washed with 70% EtOH followed by another centrifugation at 12,000*g* for 20 min. The pellet was dissolved in 50 μL Milli‐Q water. Isolated tRNA (~50 μg) was digested with one unit of nuclease P1 (Sigma, St. Louis, Missouri, USA) for 16 h at 37°C and treated with 0.5 units bacterial alkaline phosphatase for 2 h at 37°C. The hydrolysate was analyzed by high‐pressure liquid chromatography (HPLC) using a Develosil C‐30 reverse‐phase column as described elsewhere (Björk et al. 2001).

### Protein extraction and western blot

Cells were grown at 30°C to logarithmic phase (OD_600_ ~0.5). For protein extraction without trichloroacetic acid (TCA), 20 OD_600_ units of cells were resuspended in 700 μL breaking buffer (50 mmol/L Tris‐HCl [pH 7.5], 50 mmol/L NaCl, 0.2% TritonX‐100) and complete protease inhibitor cocktail (Roche Applied Science, Penzberg, Upper Bavaria, Germany, 05056489001), following the manufacturer's recommendation. Cells were broken with glass beads, and 10 μL (100 μg) of isolated total protein was loaded on SDS (sodium dodecyl sulfate) gels (Lamb et al. 1994; Fichtner et al. 2003). For protein extraction including TCA, five OD_600_ units of cells were resuspended in 500 μL breaking buffer (20 mmol/L Tris‐HCl [pH 8.0], 50 mmol/L NH_4_OAc, 2 mmol/L EDTA, and complete protease inhibitor cocktail), mixed with 500 μL ice‐cold 20% TCA, and broken with glass beads. Cell suspension was centrifuged, dissolved in 300 μL TCA–Laemmli loading buffer (Hann and Walter 1991), and 10 μL of it was loaded on SDS gel (Peter et al. 1993). During cell lysis for both methods, a FastPrep‐24 homogenizer (MP Biomedicals, Santa Ana, California, USA) was used to break the cells. Yeast anti‐Elp1p antibody recognizing the carboxyl‐terminus was designed based on previous work (Wittschieben et al. 1999) and obtained from GenScript. Anti‐GFP antibody was obtained from Roche. A 1:1000 dilution of both antibodies were used to detect Elp1p and Elp1p‐GFP, respectively. The actin and *α*‐tubulin levels were detected by using mouse anti‐Act1 antibody (Thermo Scientific, Waltham, Massachusetts, USA) and rat anti‐*α*‐tubulin antibody (Sigma Aldrich, St. Louis, Missouri, USA) at a 1:1000 dilution.

### Two‐step TAP‐tag purification of Elongator complex

Strain YSC1177‐YLR384C (Open Biosystems, Inc.) has a TAP‐tag which is fused in frame to *ELP1* at the COOH‐terminus. TAP‐tag purification was essentially as described earlier (Puig et al. 2001). Strain YSC1177‐YLR384C was cultivated in 10 mL YEPD (Yeast Extract Peptone Dextrose) over night at 30°C and 3 mL of the yeast culture was inoculated to 3 L YEPD (1× YEP, 2% glucose, 0.67 mmol/L tryptophan, and 0.33 mmol/L adenine) in a 10 L flask. The strain was grown at 30°C for 16 h to OD_600_ ~5.0. Cells were harvested by centrifugation at 5000*g* for 5 min and about 40 g cells were obtained. Cells were broken using a SPEX CertiPrep 6850 Freezer/Mill, SPEX SamplePrep, Metuchen, New Jersey, USA and resuspended in 100 mL 2× buffer A (200 mmol/L Hepes (4‐(2‐hydroxyethyl)‐1‐piperazineethanesulfonic acid) [pH 7.9], 400 mmol/L KCl, 3 mmol/L MgCl_2_, 0.5 mmol/L DTT (Dithiothreitol), 10 mmol/L NaF, 2 mmol/L sodium orthovanadate, and 0.5 mmol/L PMSF (Phenylmethanesulfonylfluoride)) and centrifuged at 25,000*g* for 30 min. The supernatant was transferred to Polyallomer centrifuge tubes (Beckman Coulter, Inc., Brea, California, USA) and centrifuged at 100,000*g* for 1 h. The supernatant was dialyzed against 1 L dialysis buffer (20 mmol/L Hepes [pH 7.9], 20% glycerol, 50 mmol/L KCl, 0.5 mmol/L EDTA (Ethylenediaminetetraacetic acid), 0.5 mmol/L DTT, 10 mmol/L NaF, 2 mmol/L sodium orthovanadate, and 0.5 mmol/L PMSF) at 4°C for 3 h. The dialyzed extract (around 70 mL) was mixed with 300 μL of a suspension containing IgG sepharose beads (GE Healthcare, Little Chalfont, United Kingdom) and 1/10 extract volume of adjusting buffer (100 mmol/L Tris‐HCl [pH 8.0], 1 mol/L NaCl, and 1% NP‐40 (Nonidet P‐40, octylphenoxypolyethoxyethanol)) in 50 mL falcon tubes. The mixture was gently rotated at 4°C for 3 h, loaded to an Econo‐Pac disposable chromatography column and eluted by gravity flow. The column was washed with 3× 10 mL cold wash buffer (10 mmol/L Tris‐HCl [pH 8.0], 150 mmol/L NaCl, and 0.1% NP‐40), then washed with 10 mL of cold TEV (tobacco etch virus) cleavage buffer (10 mmol/L Tris‐HCl [pH 8.0], 150 mmol/L NaCl, 0.1% NP‐40, 0.5 mmol/L EDTA [pH 8.0], and 1 mmol/L DTT). One milliliter cold TEV cleavage buffer containing 100 units TEV protease (Sigma) was added. The column was sealed and gently rotated at 4°C overnight. After cleavage by TEV protease, protein was eluted by gravity flow. Another column with 200 μL suspension of calmodulin beads (GE Healthcare) was equilibrated with 10 mL of cold calmodulin‐binding buffer (10 mmol/L Tris‐HCl [pH 8.0], 150 mmol/L NaCl, 0.1% NP‐40, 1 mmol/L MgAc, 1 mmol/L imidazole, 2 mmol/L CaCl_2_, and 10 mmol/L 2‐mercaptoethanol). A mixture of 3 mL cold calmodulin‐binding buffer, 3 μL of 1 mol/L CaCl_2_, and 1 mL of protein extract from the first‐step purification was applied to the column that was gently rotated at 4°C for 3 h. The column was eluted by gravity flow and washed with 4 × 10 mL cold calmodulin‐binding buffer. Finally, the target proteins were eluted by adding 5 × 200 μL cold calmodulin elution buffer (10 mmol/L Tris‐HCl [pH 8.0], 150 mmol/L NaCl, 0.1% NP‐40, 1 mmol/L MgAc, 1 mmol/L imidazole, 2 mmol/L EGTA, and 10 mmol/L 2‐mercaptoethanol). Purified protein was kept at −80°C for further analysis.

### Protein digestion and LC‐MS

Intact proteins were TMT‐0 labeled (Pierce, Waltham, Massachusetts, USA) according to the manufacturer's instructions.The labeled proteins where denatured in 6 mol/L guanidine buffer and reduced with DTT (3 mg/mL) at 75°C for 60 min followed by alkylation using iodoacetamide (15 mg/mL) for 30 min in the dark at room temperature. Reduced and alkylated proteins where digested overnight with either trypsin (Promega, Madison, Wisconsin, USA) or GluC (Roche, Penzberg, Upper Bavaria, Germany) in 50 mmol/L ammonium bicarbonate buffer (pH 8.5). The peptides were cleaned using in‐house produced stage tips (Rappsilber et al. 2003), dried, and resuspended in 0.1% trifluoroacetic acid for analysis by reverse phase liquid chromatography electrospray ionization tandem mass spectrometry (LC‐ESI‐MS/MS). Peptides where separated on a nano ACQUITY^™^ UPLC system (Waters, MA) solvent A (0.1% formic acid [FA] in water), solvent B (0.1% FA in ACN, Acetonitrile) equipped with a C18 75 μm × 100 mm reverse phase column (Waters) using a gradient of 1–30% solvent B over 90 min with a flow rate of 300 nL/min. The mass spectrometer (Waters Synapt G2 HDMS) equipped with a nanoflow electrospray ionization (ESI) interface was operated in positive ionization mode with a minimal resolution of 20,000. All data were collected in continuum mode and mass‐corrected using Glu‐fibrinopeptide B. The data were processed with Protein Lynx Global Server v.2.5.2 (Waters) and the resulting spectra were searched against Uniprot databank with *S. cerevisiae* as taxonomy filter on our in‐house MASCOT server (Matrix Science Ltd.), using a precursor tolerance of 10 ppm and a fragment tolerance of 0.1 Da.

## Results and Discussion

### Translation of Elp1p starts at the second AUG of the *ELP1* ORF

**Figure 1:**
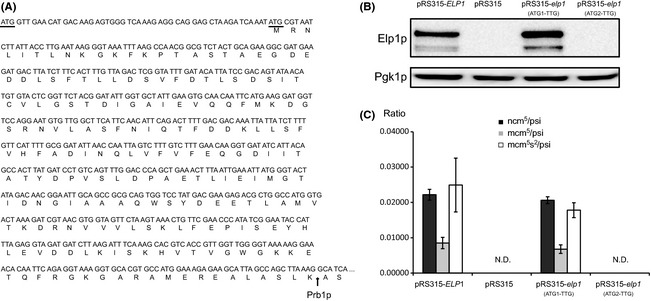
Translation of Elp1p starts at the second AUG of the *ELP1* open reading frame (ORF). (A) DNA sequence and amino acids encoded from the *ELP1* ORF up to the cleavage site by protease Prb1p. The two in‐frame 5′ ATG methionine codons are underlined. Cleavage site by Prb1p is indicated by an arrow. Identification of Prb1 and its cleavage site is presented later in Results and Discussion section. (B) Determination of Elp1p translational start site. An *elp1* null mutant (UMY3906) was transformed with plasmid pRS315, pRS315 with a wild‐type *ELP1* gene, pRS315 with an *elp1* gene having ATG_1_ mutated to TTG, or pRS315 with an *elp1* gene having ATG_2_ mutated to TTG. The expression of Elp1p was detected by anti‐Elp1p antibody. Total protein was extracted using a method not including TCA (trichloroacetic acid). (C) Levels of modified nucleosides in strains described in (B). Total tRNA was isolated from three biological replicates and levels of modified nucleosides ncm^5^U, mcm^5^U, and mcm^5^s^2^U were determined by high‐pressure liquid chromatography (HPLC). Pseudouridine (psi) was used as internal control. Error bars represent standard deviation from three biological replicates. N.D. indicates not detectable.

In the *S. cerevisiae ELP1/YLR384C* ORF there is an in‐frame ATG codon 48 nt downstream the first ATG codon (Fig. 1A). Based on comparison of closely related *Saccharomyces* species, the second but not the first ATG codon is conserved, suggesting that the start site of translation is the second ATG (Kellis et al. 2003). In order to analyze if ATG_1_ or ATG_2_ acts as the translational start codon, both codons were independently mutagenized from ATG (Met) to TTG (Leu) by oligo‐directed mutagenesis. Plasmids with either mutant derivative of the *ELP1* gene were transformed to an *elp1* null mutant (UMY3906) and the expression of Elp1p was determined by western blot. When ATG_1_ was mutated to TTG, the expression level of Elp1p was similar as wild type, whereas when ATG_2_ was mutated, no Elp1p was detected (Fig. 1B). Consistent with these findings, analysis of modified nucleosides from tRNA revealed that the plasmid with ATG_1_ mutated to TTG complemented the wobble uridine modification defect, and the plasmid with ATG_2_ mutated to TTG did not complement the tRNA modification defect (Fig. 1C). Thus, under these growth conditions, that is, exponential growth in synthetic complete media lacking leucine, AUG_2_ is the physiological translational start codon for Elp1p.

### The protease Prb1p is required for N‐terminal truncation of Elp1p

**Figure 2:**
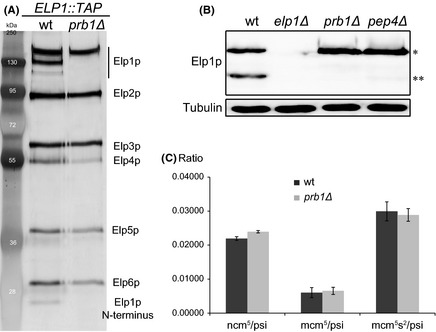
Prb1p is required for N‐terminal truncation of Elp1p. (A) Purification of Elongator complex from wild‐type (YSC1177‐YLR384C) and *prb1Δ* mutant (UMY3864) strains. The Elongator complex was purified by a two‐step TAP‐tag purification procedure. For each sample, about 2 *µ*g of purified proteins were separated on 10 % SDS‐PAGE (sodium dodecyl sulfate polyacryl amide gel electrophoresis) and visualized by silver staining. (B) Western blot of wild‐type (BY4741), *elp1Δ* (UMY3906), *prb1Δ* (YSC1053‐YEL060C), and *pep4Δ* (YSC1053‐YPL154C) strains. Total protein was extracted using a method not including TCA (trichloroacetic acid) as described in Experimental Procedures section. Elp1p was detected using an Elp1p antibody recognizing the C‐terminus of Elp1p. Single asterisk represented full‐length Elp1p and double asterisk truncated Elp1p. (C) Quantification of ncm^5^U, mcm^5^U, and mcm^5^s^2^U nucleoside levels in total tRNA isolated from wild‐type (BY4741) and *prb1Δ* mutant (YSC1053‐YEL060C) strains. Analysis of nucleosides was done by high‐pressure liquid chromatography (HPLC). The modification level is shown as the ratio between xm^5^U and pseudouridine (psi), where xm^5^U is ncm^5^U, mcm^5^U, or mcm^5^s^2^U. Error bars represent standard deviation from three biological replicates.

In order to analyze the in vivo composition of the Elongator complex, a TAP‐tagged Elp1p was expressed in strain YSC1177‐YLR384C and the complex was purified using the TAP procedure (Puig et al. 2001). Following purification, the identity of each band extracted from an SDS‐PAGE gel was determined by mass spectrometry (data not shown). Consistent with an earlier TAP purification of Elongator complex, a six‐subunit complex was obtained (Fig. 2A, left panel) (Krogan and Greenblatt 2001). Also consistent with the earlier affinity purification or western blot using human influenza HA‐tagged Elp1 protein, two major (~160  and ~140 kDa) and a minor form (~120 kDa) of Elp1p was observed (Fig. 2A, left panel) ( Krogan and Greenblatt 2001; Fichtner et al. 2003). The two major forms are full‐length Elp1p and an N‐terminal‐truncated Elp1p, whereas the minor form represents an Elp1p that is truncated at both N‐ and C‐termini (data not shown). Elongator complex is a dimeric complex containing two copies of the six‐subunit complex (Glatt et al. 2012). Therefore, the Elp1p form missing the C‐terminus is most likely copurified as part of the dimeric Elongator complex. In addition to these three forms, a fourth form of Elp1p (~26 kDa) representing the Elp1p N‐terminus was identified (Fig. 2A, left panel). As the full‐length Elp1p (~160 kDa) is processed to generate ~140 and ~26 kDa fragments, we hypothesized that an endopeptidase should be responsible for the cleavage of Elp1p.

To identify the protease required for Elp1p cleavage, we screened 95 nonessential peptidase/protease null mutants strains (Table S1) for the inability to produce truncated Elp1p. The pattern of Elp1p was determined in protein extracts from the mutants by western blot, utilizing an antibody recognizing the C‐terminus of Elp1p. Among the peptidase and protease mutants tested, we found that the *prb1Δ* and *pep4Δ* mutants showed no or reduced amounts of truncated Elp1p (Fig. 2B). Prb1p is a vacuolar protease with a complex maturation pathway that is dependent on Pep4p (Mechler et al. 1988; Moehle et al. 1989). From the *prb1* null mutant strain (UMY3864), the Elongator complex was purified by TAP‐tag affinity purification (Fig. 2A, right panel). In the absence of Prb1p, the ~140, ~120, and ~26 kDa fragments were not observed (Fig. 2A, right panel). We also observed reduced amounts of the subcomplex consisting of Elp4p, Elp5p, and Elp6p. This is not caused by the *prb1Δ*allele, rather it reflects that the TAP‐tag is located on Elp1p, making the purification more efficient for the Elp1p–Elp3p core complex and occasionally the Elp4p–Elp6p subcomplex is less efficiently purified.

In order to investigate whether loss of Prb1p influences levels of modified nucleosides, total tRNA from the *prb1Δ* (YSC1053‐YEL060C) and wild‐type (BY4741) strains was isolated and the levels of ncm^5^U, mcm^5^U, and mcm^5^s^2^U nucleosides were determined. No significant difference was observed in levels of modified nucleosides (Fig. 2C). These results show that removal of Prb1p does not influence the ability of the Elongator complex to modify wobble uridines in tRNA.

### Elp1p is cleaved between 203rd (Lys) and 204th (Ala) residues

**Figure 3:**
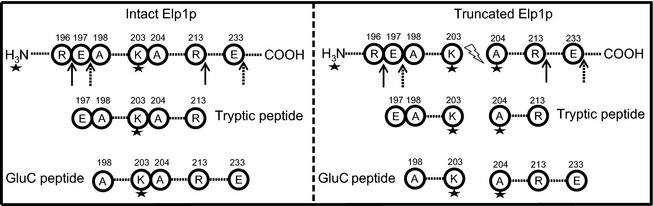
Peptides generated by trypsin or GluC treatment in amino acid regions 196–233 of the Elp1 protein. Star represents the Tandem Mass Tag (TMT) which is attached to the free N‐terminus and lysine (K). Arrows, solid, or dotted lines indicate cleavage sites of trypsin or GluC peptidases, respectively. Position of amino acid in Elp1p is indicated above. Dotted lines represent additional amino acids in Elp1p. Prb1p‐dependent cleavage site is represented by a lightning symbol. Amino acid symbols: R, arginine; E, glutamic acid; A, alanine; and K, lysine.

A short form of Elp1p was reported to be missing in about 200 amino acids in the N‐terminus (Fichtner et al. 2003). To precisely determine the Prb1p cleavage site, we purified the Elongator complex from wild‐type strain (YSC1177‐YLR384C). The purified Elongator complex (Fig. 2A, left panel), containing both full‐length (~160 kDa) and various truncated Elp1p fragments (~140, ~120, and ~26 kDa), was labeled with an amine‐reactive Tandem Mass Tag (TMT), which will attach to any free N‐terminus and to side chains of lysines (K). The benefits of TMT tagging is twofold; first, it generates distinct mass shifts that are used when identifying the peptides and second, upon fragmentation a reporter ion is generated that helps to verify that the tag is present on the peptide. The trypsin protease cleaves peptide chains mainly at the carboxyl side of lysine (K) and arginine (R) (Northrop and Kunitz 1931). However, trypsin activity on tagged lysines is greatly reduced due to the sterical hindrance of the TMT. One of the tryptic peptides generated from an intact Elp1p stretched from glutamic acid (E) at position 197 to arginine (R) at position 213 with a TMT‐tagged K at position 203 (Figs. 3 and S1A). In the same region, two tryptic peptides were generated from the truncated Elp1p, E197 – K203 with a TMT‐tagged K at the peptide C‐terminus (Figs. 3 and S1B), and A204 – R213 with a TMT on the peptide N‐terminus (Figs. 3 and S1C). In order to verify the trypsin‐digested sample, another batch of sample was digested using endopeptidase GluC in a buffered solution at pH 8.5 which makes the enzyme strongly favor cleavage after glutamic acid (E) over aspartic acid (D) (Birktoft and Breddam 1994). A GluC peptide generated from the intact Elp1p stretched from A198 – E233 with a TMT‐tagged K at position 203 (Fig. 3 and S1D), while two peptides generated from the truncated Elp1p were identified, an A198 – K203 with a TMT‐tagged K at the peptide C‐terminus (Fig. 3, spectra not shown), and an A204 – E233 with a TMT on the peptide N‐terminus (Fig. 3 and S1E). For both the trypsin‐ and GluC‐digested samples, we could with high confidence identify a truncation site between position 203 and 204 (Figs. S1B, S1C, S1E and 1). Although we could find the C‐terminal end of the truncated N‐terminus of Elp1p (~26 kDa) in both samples, only the identification from the trypsin sample was unambiguous. In addition, the N‐terminal of the larger fragment (~140 kDa) was identified with high confidence in both samples. Thus, we conclude that the two major forms of Elp1p in the TAP‐tag purification represent full‐length Elp1p and a shorter form of Elp1p cleaved between K203 and A204, hereafter called Elp1p_Del N‐term_.

### The Elp1p_Del N‐term_ is not active in wobble uridine tRNA modification

**Figure 4:**
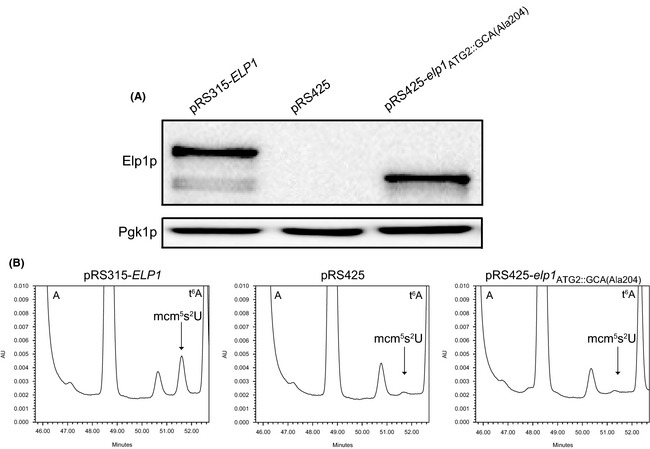
Expression of N‐terminal truncated Elp1p does not complement the tRNA modification defect of an *elp1Δ* strain. (A) Western blot showing the expression of Elp1p_Del N‐term._ An *elp1* null mutant was transformed with pRS315‐*ELP1*, pRS425 empty control vector, and pRS425‐*elp1*_ATG2::GCA(Ala204)_, respectively. Proteins were isolated by a protein extraction method without TCA (trichloroacetic acid) and in western blot an Elp1p antibody was utilized to detect Elp1p. (B) Presence of modified nucleoside mcm^5^s^2^U in strains described in (A). Total tRNA was isolated, digested to nucleosides, and analyzed by high‐pressure liquid chromatography (HPLC). The arrow indicates where the modified nucleoside 5‐methoxycarbonylmethyl‐2‐thiouridine (mcm^5^s^2^U) should migrate. The small peaks at this position (middle and right panels) represent an unrelated compound with a spectrum different from mcm^5^s^2^U. Peaks representing adenosine (A) and *N*6‐threonylcarbamoyladenosine (t^6^A) are indicated.

To investigate if the Elp1p_Del N‐term_ is functional in wobble uridine tRNA modification, it was expressed in an *elp1* null mutant. Both low and high copy plasmids were constructed where the *elp1* gene encoding the Elp1p_Del N‐term_ (starting from A204) was cloned in frame with the AUG_2_ translational start codon. In order to obtain a similar expression level as from the wild‐type *ELP1* gene, Elp1p_Del N‐term_ was expressed from a high copy vector (Fig. 4A). Total tRNA from the *elp1* null mutant expressing the Elp1p_Del N‐term_ short form was isolated and analyzed for the presence of modified nucleosides. Expression of the Elp1p_Del N‐term_ did not complement the wobble uridine modification defect of an *elp1* null mutant (Fig. 4B), indicating that Elp1p_Del N‐term_ is not active in tRNA modification.

### Truncated Elp1p is a preparation artifact during sample preparation

**Figure 5:**
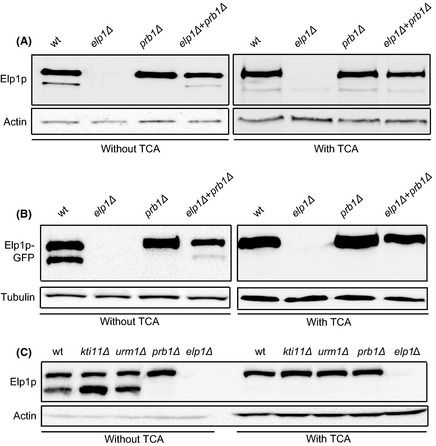
N‐terminal truncation of Elp1p is a preparation artifact. (A) Cell pellets from wild‐type (BY4741), *elp1Δ* (UMY3906), *prb1Δ* (YSC1053‐YEL060C), and a mixed cell pellet where half of the cells was from strain *elp1Δ* (UMY3906) and the other half from strain *prb1Δ* (YSC1053‐YEL060C) were collected from exponential growing cells. Each pellet including the mixed pellet represent the same total amount of cells. Protein was extracted from cells pellets in the absence (left panel) or presence of trichloroacetic acid (TCA) (right panel). Elp1p was detected by western blot using anti‐Elp1p antibody. (B) Cell pellets from strains *ELP1::GFP* (95700‐YLR384C), *elp1Δ* (UMY 3906), and *ELP1::GFPprb1Δ* (UMY3930) and a mixed cell pellet were prepared and used for protein extraction as described in (A). Elp1‐GFP fusion protein was detected by western blot using anti‐GFP antibody. (C) Cell pellets from wild‐type (BY4741), *kti11Δ* (UMY3771), *urm1Δ* (UMY3551), *prb1Δ* (YSC1053‐YEL060C), and *elp1Δ* (UMY3906) strains were protein extracted in the absence of TCA (left panel) or in the presence of TCA (right panel). Elp1p was detected by western blot using anti‐Elp1p antibody. As loading control, actin or tubulin were used.

An intact Elongator complex was purified in the absence of Prb1p and formation of modified wobble uridines was not influenced in the *prb1* null mutant (Fig. 2A and C). In addition to these observations, expression of the Elp1p_Del N‐term_ did not complement the tRNA modification defect of an *elp1* null mutant strain (Fig. 4B). Therefore, we considered the possibility that the cleavage of Elp1p is an artifact taking place during sample preparation. To address this question, exponentially growing wild‐type (BY4741), *elp1Δ* (UMY3906), and *prb1Δ* (YSC1053‐YEL060C), strains were used to prepare cell pellets. One sample was also prepared where equal amounts of cell pellet from *elp1Δ* (UMY3906) and *prb1Δ* (YSC1053‐YEL060C) cultures were mixed. Proteins from wild‐type, *elp1Δ*,* prb1Δ*, and the mixed *elp1Δ/ prb1Δ* cell pellets were extracted. In the mixed *elp1Δ/ prb1Δ* cell pellet, Elp1p is only present in the *prb1Δ* strain and Prb1p is only present in the *elp1Δ* strain. Therefore, the appearance of truncated Elp1p would imply an in vitro endopeptidase cleavage by Prb1p originating from the *elp1Δ* strain occurring during sample preparation. Since a truncated Elp1p was detected from the mixed *elp1Δ/ prb1Δ* cell pellet (Fig. 5A, left panel), truncation of Elp1 occurred in vitro during sample preparation. To minimize a possible in vitro endopeptidase cleavage of Elp1p during sample preparation and to investigate whether truncated Elp1p could be observed in vivo in the wild‐type strain (BY4741), a protein extraction method including TCA was used (Peter et al. 1993). In this method, the extracted proteins are precipitated with TCA immediately after cell lysis and denatured. In the previous study, where truncated Elp1p was observed, no TCA was included in the protein extraction (Fichtner et al. 2003). However, when TCA was included and Elp1p was detected by the anti‐Elp1p antibody, an unspecific band occasionally appeared in the *elp1Δ* strain with almost the same size as truncated Elp1p (Fig. 5A, right panel). This inconsistency made it difficult to determine whether the Elp1p N‐terminal truncation takes place in vivo or in vitro. To solve this problem, we made use of strains harboring Elp1‐GFP protein fusions generating a slower migrating product. Thus, we used the *ELP1‐GFP* (95700‐YLR384C) strain and constructed an *ELP1‐GFP prb1Δ* derivative (UMY3930). The experiment described above was repeated using strains *ELP1‐GFP* (95700‐YLR384C), *ELP1‐GFP prb1Δ* (UMY3930), and *elp1Δ* (UMY3906). The Elp1‐GFP protein was detected with an anti‐GFP antibody, which did not give any unspecific signal at the position of truncated Elp1p. No truncated form of Elp1p was detected in pellets from wild‐type or mixed *elp1Δ/ prb1Δ* cell pellets using the TCA method (Fig. 5B, right panel), whereas the truncated form was observed when TCA was excluded (Fig. 5B, left panel). This result shows that the Elp1p N‐terminal truncated form is generated during sample preparation.

It was previously shown that more truncated Elp1p was observed in protein extracts from *kti11Δ* or *urm1Δ* mutants than from a wild‐type strain (Fichtner et al. 2003). We considered that this observation might also be a result of a protein preparation artifact. To test this hypothesis, a western blot was performed to determine the relative amounts of full‐length and truncated Elp1p in these mutants by extracting proteins in the absence or presence of TCA (Fig. 5C). When TCA was not included during protein extraction, more truncated Elp1p was observed in the *kti11Δ* and *urm1Δ* mutants compared to the wild‐type strain (Fig. 5C, left panel). This observation is similar as the previous finding (Fichtner et al. 2003). However, no truncated Elp1p was detected in the mutants when TCA was included (Fig. 5C, right panel). These data support that appearance of truncated Elp1p is a preparation artifact. The reason why more truncated Elp1p was observed when TCA was excluded during protein extraction might be that Kti11p and Urm1p protect Elp1p from cleavage of Prb1p.

Apparently, in the presence of Prb1p without using TCA during protein extraction, truncation of Elp1p is prone to occur. Prb1p is a vacuolar protease and the vacuole serves as a compartment to degrade cytoplasmic proteins and organelles by autophagy under nitrogen starvation (Li and Kane 2009). Therefore, it will not be surprising that under nitrogen starvation Elp1p is delivered to vacuole, trimmed by Prb1p, and degraded.

Although both protein extraction buffers contain a protease inhibitor cocktail, it is obviously not enough to inhibit the action of protease Prb1p. Thus, to be sure to circumvent cleavage or degradation of proteins to be studied, alternative protein extraction methods should be used to avoid misinterpretations of the data.

## Conflict of Interest

None declared.

## Supporting information


**Figure S1.** MS/MS fragmentation spectra of peptides generated by trypsin or GluC treatment in the 213–250 amino acid region of Elp1p. As starting material, purified Elongator complex from wild‐type strain was used. (A) Trypsin‐generated peptide EALASLK¹ASGLVGNQLR, ion score 96, *E*‐value = 1.1e^−9^. (B) Trypsin‐generated peptide EALASLK¹, ion score 46, *E*‐value = 2.9e^−4^. (C) Trypsin‐generated peptide ¹ASGLVGNQLR, ion score 58, *E*‐value = 1.9e^−5^. (D) GluC‐generated peptide ALASLK¹ASGLVGNQLRDPTMPYMVDTGDVTALDSHE, ion score 150, *E*‐value = 2.7e^−14^. (E) GluC‐generated peptide ¹ASGLVGNQLRDPTMPYMVDTGDVTALDSHE, ion score 57, *E*‐value = 4.8e^−6^. Ion score: on average, individual ions scores >30 indicate identity or extensive homology (*P* < 0.05). *E*‐value: expectation value for the peptide match, the number of times expected to obtain an equal or higher score, purely by chance. ¹ denotes the position of the Tandem Mass Tag (TMT).

**Table S1**. Peptidase/protease mutants screened for cleavage of Elp1p.
