# Supplementary material for: Yeast Elongator protein Elp1p does not undergo proteolytic processing in exponentially growing cells
Source: Microbiologyopen. 2015 Sep 25;4(6):867–78. doi: 10.1002/mbo3.285 (PMC4694139; doi:10.1002/mbo3.285)
Supplement: Supplementary file 2 — Table S1. Peptidase/protease mutants screened for cleavage of Elp1p. [file MBO3-4-0867-s002.docx]

**Supplementary Information**

Table S1 Peptidase/protease mutants screened for cleavage of Elp1p.

| MATa *his3Δ1 leu2Δ0 ura3Δ0 met15Δ0 X::kanMX* (Open bio-systems, YSC1053-YEL060C) | | | | |
| --- | --- | --- | --- | --- |
| *aap1* | *esp1* | *oma1* | *ste13* | *uip5* |
| *afg1* | *fra1* | *otu1* | *ste23* | *vps70* |
| *afg3* | *hsp31* | *otu2* | *ste24* | *wwm1* |
| *ape2* | *icp55* | *pcp1* | *tma108* | *ybr139w* |
| *ape3* | *imp1* | *pep4* | *ubp1* | *ydr415c* |
| *arx1* | *imp2* | *pim1* | *ubp2* | *yfr018c* |
| *atg4* | *kex1* | *prb1* | *ubp3* | *yhr113w* |
| *atp23* | *kex2* | *prc1* | *ubp5* | *yil108w* |
| *axl1* | *lap2* | *prd1* | *ubp6* | *ykl100c* |
| *bar1* | *lap3* | *pre9* | *ubp7* | *yme1* |
| *bre5* | *lap4* | *qri7* | *ubp8* | *yol057w* |
| *cps1* | *map1* | *rbd2* | *ubp9* | *yps1* |
| *cym1* | *map2* | *rce1* | *ubp11* | *yps3* |
| *dap2* | *mca1* | *rim13* | *ubp12* | *yps5* |
| *doa4* | *mgr1* | *rri1* | *ubp13* | *yps6* |
| *dug1* | *mgr3* | *rrt12* | *ubp14* | *yps7* |
| *dug2* | *mkc7* | *sno4* | *ubp15* | *ysp3* |
| *dug3* | *nma111* | *spc1* | *ubp16* | *yta12* |
| *ecm14* | *oct1* | *spc2* | *uip4* | *yuh1* |

Figure S1. MS/MS Fragmentation spectra of peptides generated by trypsin or GluC treatment in the 213 to 250 amino acid region of Elp1p. As starting material, purified Elongator complex from wild type was used. (A) Trypsin generated peptide EALASLK¹ASGLVGNQLR, Ion Score 96, E-value: 1.1e^-9^ (B) Trypsin generated peptide EALASLK¹, Ion Score 46, E-value: 2.9e^-4^ (C) Trypsin generated peptide ¹ASGLVGNQLR, Ion Score 58, E-value: 1.9e^-5^ (D) GluC generated peptide ALASLK¹ASGLVGNQLRDPTMPYMVDTGDVTALDSHE, Ion Score 150, E-value: 2.7e^-14^ (E) GluC generated peptide ¹ASGLVGNQLRDPTMPYMVDTGDVTALDSHE, Ion Score 57, E-value: 4.8e^-6^. Ion Score: On average, individual ions scores > 30 indicate identity or extensive homology (p<0.05). E-value: Expectation value for the peptide match, the number of times expected to obtain an equal or higher score, purely by chance. ¹ denotes the position of the TMT tag
